# Using medical university websites for health education about COVID-19; an effective solution for public education during a pandemic

**DOI:** 10.1186/s41043-023-00417-y

**Published:** 2023-07-28

**Authors:** Lina Cai, Jiwei Han, Zahra Aghalari, Hans-Uwe Dahms‬

**Affiliations:** 1 Department of Pediatrics, North China University of Science and Technology Affiliated Hospital, Tangshan, 063000 Hebei China; 2 School of Management, North China University of Science and Technology, Tangshan, 063000 Hebei China; 3 Social Determinants of Health Research Center, Health Research Institute, Babol University of Medical Sciences, Babol, I.R. of Iran; 4 Department of Biomedical Science and Environmental Biology, College of Life Science, Kaohsiung Medical University, Kaohsiung, Taiwan, ROC; 5 Research Center for Environmental Medicine, KMU - Kaohsiung Medical University, Kaohsiung, 80708 Taiwan, ROC

**Keywords:** Medical university, Website, Health education, COVID-19, Epidemiological prevention

## Abstract

**Background:**

Medical universities use their websites to teach, research, and promote a culture of health. Therefore, this study aimed to evaluate the performance of medical universities in terms of health information and education regarding COVID-19 by surveying the website of Iranian medical universities.

**Methods:**

This descriptive-analytical study was conducted in June to August 2020 on the websites of medical universities in three categories of universities (type 1, type 2 and type 3). The information of this study was collected from medical universities located in the east, west, north, south and center of Iran. Data were collected according to a checklist. The checklist contained 3 sections; the first part with 8 components regarding general information of the university websites, the second part with 11 components regarding the information and news related to the coronavirus and the third part with 12 components regarding the content of personal health education and environmental health for the prevention of coronavirus. To determine the status of each website in the two areas of health information and education, websites were divided into three categories based on scores (poor, average and good). Data were analyzed by chi-square.

**Results:**

In this study, 1118 web pages related to 48 Iranian universities of medical sciences were reviewed, where 19 were type 1 universities, 21 type 2 universities, and 8 type 3 universities. The mean scores of the websites regarding the information and news related to the coronavirus (8.54 ± 1.750) and the mean scores of the websites regarding the personal and environmental health education related to coronavirus (10.96 ± 1.148) were in a favorable and positive condition. The ranking of medical universities by type showed that the scores in the two areas of health information and education about the coronavirus were in good condition and none of the universities were in bad condition. Chi-square showed that the information status and news related to the coronavirus had a significantly positive relationship with the type of medical universities (χ^2^ = 10.343, p = 0.006).

**Conclusions:**

The results of this study showed that type1 and type 2 and 62.5% of type 3 medical universities were in good condition in terms of total scores in the two areas of health information and education about coronavirus and none of the universities were in a bad situation. It is suggested that the website of medical universities can serve as a reliable and appropriate source of information not only for academics and students but also for the general public.

## Background

Since December 2019, cases of severe respiratory infections have been reported in Wuhan, China, which were caused and termed the coronavirus or COVID-19 [1]. On January 4, 2020, the first death cases caused by coronavirus were reported in China.

Coronavirus positive cases were then rapidly reported from Thailand, Japan, South Korea and the United States throughout January 2020. Person-to-person transmission of the coronavirus and transmission to healthcare workers spread rapidly and caused a most devastating epidemy to mankind [2, 3]. The coronavirus was transmitted to Iran in 2019. Iran used all facilities such as health, scientific, research, and media facilities to prevent and control the coronavirus. During the outbreak of coronavirus in Iran, various audio and visual media provided news related to the coronavirus. The media sought to reduce public awareness of how to prevent coronavirus and stress. Among the various media, cyberspace and web pages were an important source for conveying health news and concepts to the public [4, 5]. Websites were designed to provide the information for researchers and to share information resources, knowledge and personal experiences [6]. Websites are a very complex type of information resources that are produced by different people and searched by different users [7]. An interested audience can use the websites of education and research, entertainment and recreation, economic and commercial development [8, 9].

Among the various websites, the websites of medical universities are thought to be of great importance as a reliable source to conveying health news and concepts to the public [10]. The websites of medical universities, like other websites, provide a structured collection of data that is displayed in the form of texts, graphic images, photos, videos, and audios to provide a variety of content and as a multimedia database, websites are considered as a suitable tool for introducing scientific, research, educational and therapeutic activities [11, 12].

Medical universities use their websites to teach, research, and promote a culture of health. Since the outbreak of the coronavirus in Iran, medical universities provided information and preventive training on the capacity of their websites in various sections, such as the hospitals website and the health deputy website [13, 14]. Since February 2019, when the outbreak of coronavirus in Iran was confirmed, the websites of medical universities were used as an information tool for professors, students and staff of medical universities. Some ordinary people in the community used the websites of Universities of Medical Sciences to find information about the prevalence of coronavirus in their city or province or to find suggestions for health education. The coronavirus provided a serious threat to the world and Iran, and according to experts, in autumn and winter there was the possibility of simultaneous outbreaks of influenza and coronavirus. For this reason the officials of medical universities were expected to pay more attention to the evaluation of university websites by identifying their strengths and weaknesses in an effort to provide news and educational contents about the coronavirus. Therefore, this study aimed to evaluate the performance of medical universities in terms of health information and education regarding COVID-19 by surveying the website of Iranian medical universities.

## Methods

### Study design and population

**Fig. 1:**
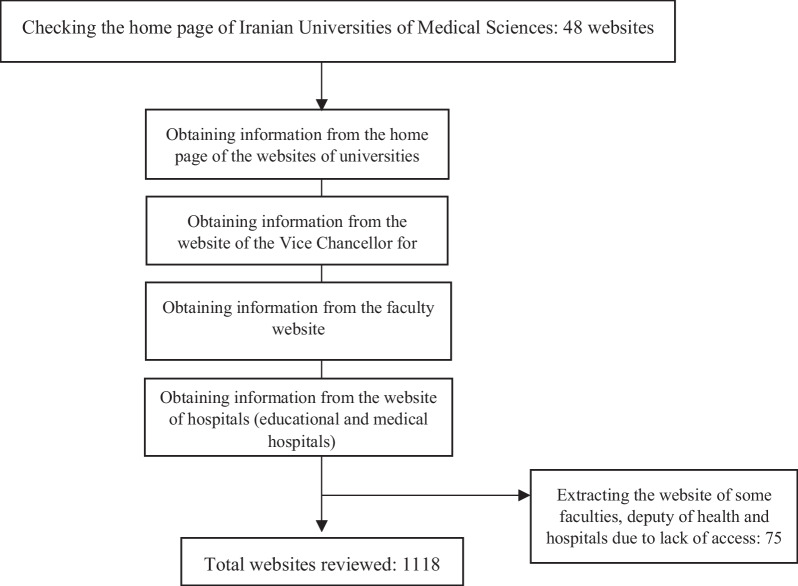
Flowchart of methodological approaches of the study

This descriptive-analytical study was conducted in June to August 2020 on the websites of medical universities of Iran. The information of this study was collected from medical universities located in the east, west, north, south and center of Iran. In the present study, the websites of Iranian universities of medical sciences were divided into three categories (type 1, type 2 and type 3 universities). The ranking of medical universities was based on indicators such as research infrastructure, student research and ethics in medical research and indicators in the field of science production. Universities that were in good condition in terms of these indicators were ranked first (type 1), universities with average and poor status were ranked second (type 2) and third (type 3), respectively. There are 48 universities of medical sciences in Iran. For this research, we first entered the main page of the website of 48 universities. Then, for each university, we checked the website pages of the faculties, the website of the Vice Chancellor for Health and the website of the educational centers (hospitals) affiliated to each university. In total, 1118 websites from 48 universities of medical sciences were reviewed (Fig. 1).

### Data collection techniques and procedures

Data were collected according to a checklist [15–17]. The checklist contained three sections: the first part with 8 components regarding general information of the university websites, the second part with 11 components regarding the information and news related to coronavirus and the third part with 12 components regarding the content of personal health education and environmental health for the prevention of coronavirus. The validity of the checklist was confirmed by the opinions of 2 information science experts and 3 health experts. To assess each of the components of the checklist, two researchers of this study referred to the website of medical universities, the website of the faculties, the website of the Vice Chancellor for Health and the website of the educational centers (hospitals) of each university and reviewing the different components. For the presence of component in the websites, a score of one and for the absence of the components in the websites, a score was zero. Likert scoring methods were used to respond to the checklists. For each sector, the scores below 49% were poor, 50% considered as average and over 51% were considered as good [18].

In the checklist for news and information about the coronavirus, the minimum and maximum scores of the websites were 0 to 11. Regarding the status of information and news of each website, the scores obtained were reported within three levels: below 3 (poor), 4 to 7 (medium) and 8 to 11 (good).

In the personal and environmental health education checklist, the minimum and maximum scores for each website were zero to 12. Regarding the status of the personal and environmental health education of each website, the scores obtained were reported at three levels: below 4 (poor), 5 to 8 (medium) and 9 to 12 (good).

The scores of the two sections of information and news, personal and environmental health education were zero to 23 for each of the websites. The scores obtained were reported for three levels: below 7 (poor), 8 to 15 (medium) and 16 to 23 (good).

### Data analysis

Data were analyzed by SPSS software version 22, descriptive and analytical statistics. Relative frequency and percentage were used for data analysis in the descriptive statistics section, and chi-square test was used for statistical analysis. P < 0.05 was considered as statistically significant.

## Results

In this study, 1118 web pages of 48 Iranian universities of medical sciences related to health departments, faculties and hospitals of Iranian universities of medical sciences. They belonged to type 1 universities [19], type two universities [21], and type three universities [8].

The mean scores of the websites regarding the information and news related to the coronavirus were in positive condition (8.54 ± 1.750). The highest percentage was related to component 6 (Ability to search for phrases related to the coronavirus). The highest mean scores were related to components 6,4 and 5 regarding the search for terms related to coronavirus. The lowest mean score was for component 2 (linking to WHO resources and subject databases for information on coronavirus) (Table 1).

**Table 1:** Scores of Iranian medical universities websites regarding information and news related to the coronavirus

Checklist components	Yes	No	Mean ± SD
1. Link to news sites and the website of the Ministry of Health to receive information about the coronavirus	29 (60.4)	19 (39.6)	0.60 ± 0.494
2. Links to the resources and website of the World Health Organization for information about the coronavirus	26 (54.2)	22 (45.8)	0.54 ± 0.504
3. Links to online newspaper and journal websites for information about the coronavirus	28 (58.3)	20 (41.7)	0.58 ± 0.498
4. Ability to easily search about the coronavirus	45 (93.8)	3 (6.2)	0.94 ± 0.245
5. Ability to advanced search about the coronavirus	45 (93.8)	3 (6.2)	0.94 ± 0.245
6. Ability to search for phrases related to the coronavirus	46 (95.8)	2 (4.2)	0.96 ± 0.202
7. Latest news about coronavirus	40 (83.3)	8 (16.7)	0.83 ± 0.377
8. Coronavirus notifications	41 (85.4)	7 (14.6)	0.85 ± 0.357
9. News about medical services provided in universities and hospitals regarding coronavirus	43 (89.6)	5 (10.4)	0.90 ± 0.309
10. News about the help of philanthropists regarding the coronavirus	29 (60.4)	19 (39.6)	0.60 ± 0.494
11. News about the morbidity and mortality of coronavirus	38 (79.2)	10 (20.8)	0.79 ± 0.410
Total mean			8.54 ± 1.750

The mean scores of the websites regarding the personal and environmental health education related to the coronavirus were generally favorable and positive (10.96 ± 1.148). The highest mean scores were related to questions 3, 4, 8, 9 and 10 regarding education on personal hygiene such as using masks and gloves, disinfectants and social distancing. The lowest mean scores were related to question 7 (hygienic disposal of solid waste produced by coronavirus) and question 6 (food hygiene) (Table 2).

**Table 2:** Scores of Iranian medical university websites regarding personal and environmental health education related to the coronavirus

Checklist components	Yes	No	Mean ± SD
1. Information about the function of the coronavirus in the body	41 (85.4)	7 (14.6)	0.85 ± 0.357
2. Information on how to protect high-risk people (the elderly, pregnant women and people with underlying diseases)	42 (87.5)	6 (12.5)	0.88 ± 0.334
3. Information about hand washing	48 (100)	0	1 ± 0
4. Information about different types of masks	48 (100)	0	1 ± 0
5. Information about different types of disinfectants	44 (91.7)	4 (8.3)	0.92 ± 0.279
6. Information about food hygiene	38 (79.2)	10 (20.8)	0.79 ± 0.410
7. Information about hygienic disposal of waste produced by the coronavirus (masks and gloves)	37 (77.1)	11 (22.9)	0.77 ± 0.425
8. Information about disinfect clothing and various items (cell phone, bag, glasses, etc.)	48 (100)	0	1 ± 0
9. Information about social distancing	48 (100)	0	1 ± 0
10. Information about system 123 coronavirus, www.123corona.com	48 (100)	0	1 ± 0
11. Information about Corona test sites, test.corona.ir	42 (87.5)	6 (12.5)	0.88 ± 0.334
12. Information about attention to hygiene in guilds	42 (87.5)	6 (12.5)	0.88 ± 0.334
Total mean			10.96 ± 1.148

Chi-square showed that the performance of Type1 and Type2 universities in terms of information and news on the coronavirus and personal and environmental health education was better than that of Type3 medical universities. Chi-square showed that the ability to search easily for coronavirus had a significant relationship with the type of medical universities (p = 0.046). Type1 universities of medical sciences were 100%, Type2 universities of medical sciences were 95.2% and Type3 universities of medical sciences were up to 75% capable of simple search for coronavirus. In 84.2% of the websites of Type 1 universities of medical sciences, in 95.2% of the websites of Type 2 universities of medical sciences, in 50% of the websites of Type 3 universities of medical sciences, the latest news of coronavirus has been published. Chi-square showed that significant relationship between coronavirus news and type of medical universities (p = 0.014). Chi-square showed that the publication of morbidity and mortality of coronavirus on the university website was significantly related to the type of medical universities (p = 0.056) (Table 3).

**Table 3:** Total scores of Iranian medical university websites regarding information and news and education on personal and environmental health about coronavirus

Checklist components	Level of Iranian Universities of Medical Sciences						χ^2^	P-value
	Type 1		Type 2		Type 3			
	Yes	No	Yes	No	Yes	No		
*Information and news about coronavirus*								
1. Link to news sites and the website of the Ministry of Health to receive information about the coronavirus	12 (63.2)	7 (36.8)	15 (71.4)	6 (28.6)	2 (25)	6 (75)	5.321	0.070
2. Links to the resources and website of the World Health Organization for information about the coronavirus	12 (63.2)	7 (36.8)	11 (52.4)	10 (47.6)	3 (37.5)	5 (62.5)	1.541	0.463
3. Links to online newspapers and journal websites for information about the coronavirus	12 (63.2)	7 (36.8)	13 (61.9)	8 (38.1)	3 (37.5)	5 (62.5)	1.721	0.423
4. Ability to search easily about the coronavirus	19 (100)	0	20 (95.2)	1 (4.8)	6 (75)	2 (25)	6.146	0.046
5. Ability to advanced search about the coronavirus	17 (89.5)	2 (10.5)	21 (100)	0	7 (87.5)	1 (12.5)	2.526	0.238
6. Ability to search for phrases related to the coronavirus	18 (94.7)	1 (5.3)	21 (100)	0	7 (87.5)	1 (12.5)	2.362	0.307
7. Latest news about coronavirus	16 (84.2)	3 (15.8)	20 (95.2)	1 (4.8)	4 (50)	4 (50)	8.553	0.014
8. Coronavirus notifications	16 (84.2)	3 (15.8)	19 (90.5)	2 (9.5)	6 (75)	2 (25)	1.151	0.563
9. News about medical services provided in universities and hospitals regarding coronavirus	18 (94.7)	1 (5.3)	19 (90.5)	2 (9.5)	6 (75)	2 (25)	2.382	0.304
10. News about the help of philanthropists regarding the coronavirus	12 (63.2)	7 (36.8)	14 (66.7)	7 (33.3)	3 (37.5)	5 (62.5)	2.160	0.340
11. News about the morbidity and mortality of coronavirus	15 (78.9)	4 (21.1)	19 (90.5)	2 (9.5)	4 (50)	4 (50)	5.775	0.056
*Education on personal and environmental health about coronavirus*								
12. Information about the function of the coronavirus in the body	18 (94.7)	1 (5.3)	17 (81)	4 (19)	6 (75)	2 (25)	2.358	0.308
13. Information on how to protect high-risk people (the elderly, pregnant women and people with underlying diseases)	17 (89.5)	2 (10.5)	19 (90.5)	2 (9.5)	6 (75)	2 (25)	1.381	0.501
14. Information about hand washing	19 (100)	0	21 (100)	0	8 (100)	0	–	–
15. Information about different types of masks	19 (100)	0	21 (100)	0	8 (100)	0	–	–
16. Information about different types of disinfectants	18 (94.7)	1 (5.3)	19 (90.5)	2 (9.5)	7 (87.5)	1 (12.5)	0.455	0.796
17. Information about food hygiene	17 (89.5)	2 (10.5)	17 (81)	4 (19)	4 (50)	4 (50)	5.391	0.068
18. Information about hygienical disposal of waste produced by the coronavirus (masks and gloves)	14 (73.7)	5 (26.3)	16 (76.2)	5 (23.8)	7 (87.5)	1 (12.5)	0.625	0.732
19. Information about disinfect clothing and various items (cell phone, bag, glasses, etc.)	19 (100)	0	21 (100)	0	8 (100)	0	–	–
20. Information about social distancing	19 (100)	0	21 (100)	0	8 (100)	0	–	–
21. Information about system 123 Coronavirus, www.123corona.com	19 (100)	0	21 (100)	0	8 (100)	0	–	–
22. Information about Corona test sites, test.corona.ir	18 (94.7)	1 (5.3)	18 (85.7)	3 (14.3)	6 (75)	2 (25)	2.114	0.348
23. Information about attention to hygiene in guilds	17 (89.5)	2 (10.5)	19 (90.5)	2 (9.5)	6 (75)	2 (25)	1.381	0.501

The highest scores about information and news about coronavirus were related to type 2 universities (90.5%) and the lowest were related to type 3 universities (5.37%).The highest scores about personal and environmental health related to coronavirus were related to type 1 and 3 universities (100%) and the lowest were related to type 2 universities (90.5%) (Table 4).

**Table 4:** Ranking of websites of Iranian universities of medical sciences about information and news and education on personal and environmental health related to coronavirus

Levels	Classification	Level of Iranian Universities of Medical Sciences			Total
		Type 1	Type 2	Type 3	
Scores about information and news about coronavirus	Medium 4–7	3 (15.8)	2 (9.5)	5 (62.5)	10 (20.8)
	Good 8–11	16 (84.2)	19 (90.5)	3 (37.5)	38 (79.2)
Scores about personal and environmental health related to coronavirus	Medium 5–8	0	2 (9.5)	0	2 (4.2)
	Good 9–12	19 (100)	19 (90.5)	8 (100)	46 (95.8)
Total scores in the two areas of health information and education	Medium 8–15	0	0	3 (37.5)	3 (6.2)
	Good 16–23	19 (100)	21 (100)	5 (62.5)	45 (93.8)

100% of Type1 and Type2 universities and 62.5% of Type 3 medical universities were in good condition in terms of total scores in the two areas of health information and education about the coronavirus, and none of the universities were in bad condition. Chi-square results showed that there was a significant relationship between medical university type and classification of scores regarding the information and news related to the coronavirus (χ^2^ = 10.343, p = 0.006). (Table 4).

## Discussion

In this study, the lowest mean score was for linking the university websites to the World Health Organization website for information on coronavirus. The highest mean score was related to the search for terms related to coronavirus on the websites of universities. The mean scores of websites regarding the information and news related to coronavirus were favorable and positive (8.54 ± 1.750). Therefore, based on the findings of the present study, scientific websites are a suitable tool for health education. In a study by Hamzehei et al. (2018), it was reported that reliable scientific websites such as health and medical websites can be used for health education about diseases[19]. In a study by Gill et al. (2013), showed that social media technologies and websites have the potential to deliver safe and effective health education[20]. In a study by Valizadeh-Haghi et al. (2021), showed that websites can be used for health education to prevent diseases, but their scientific content needs to be verified and validated[21]. The Ministry of Health must use all the information facilities, such as the website of the universities of medical sciences, to inform and publish the correct news about coronavirus among people. Linking the websites of medical universities to world-renowned websites such as the World Health Organization website can be a way to raise public awareness and create the right attitude towards the coronavirus. Therefore, it is suggested the main page of the website of Iranian universities of medical sciences should be linked to the WHO website and other reliable sources worldwide.

The mean scores of the websites regarding personal and environmental health education related to the coronavirus were favorable (10.96 ± 1.148). The highest mean scores were related to education about personal hygiene such as using masks and gloves, disinfectants and social distancing. The lowest mean scores were related to hygienic disposal of solid waste produced by coronavirus and food hygiene. In a study by Zarghani et al. (2017) with the aim of determining the services provided on the website of the central libraries of medical universities showed that 77.8% of the websites offered "library usage training" [22]. In recent years, the Internet and cyberspace have been used extensively in education and change of behavior related to information-gain and learning [23]. For example, a study conducted at Hong Kong University on online search for health information and internet usage patterns reported that the main health information used by users was in the four areas of health, treatment, family health and issues that are difficult to talk about. The results from the University of Hong Kong showed that those who frequently use the internet and websites for health information are more likely to use websites for day-to-day decisions [24]. The results of this part of our study indicate that the use of cyberspace and websites have benefits such as increasing awareness without having to leave home in the prevalence of coronavirus, easy access to information, high speed of information dissemination, access to information at low cost. It should be noted that the use of fake websites to obtain information related to disease and health can lead to harm to physical and mental health. Therefore, reputable websites such as the websites of medical universities and reputable medical science journals should be introduced to users.

Type1 and Type2 universities (100%) and Type3 (62.5%) of medical universities showed good conditions in terms of total scores in the two areas of health information and education about the coronavirus, and none of the universities were in bad conditions. In a study by Hamidi et al. (2016) with the aim of evaluating the websites of Iranian universities of medical sciences in terms of attention to content and technical characteristics showed that type one and type two universities were better and type three universities had the lowest levels of compliance [6]. Shabankareh et al. (2016) reported that the medical universities in type three should pay attention to the production of content [25]. The results of this part of the study in line with other studies indicate the unfavorable situation of type three universities. The inadequacy of the situation of Type 3 universities in the two areas of health information and education about the coronavirus can be explained with the limited facilities of Type3 universities of medical sciences compared to Type1 and Type2 universities. Therefore, authorities should pay attention to Type3 universities of medical sciences, which are tertiary universities and are located in deprived areas of the country.

## Conclusion

The results of this study showed that type1, type2 and 62.5% of type3 medical universities were in good conditions in terms of total scores in the two areas of health information and education about coronavirus and none of the universities was actually in an unacceptable situation. The use of cyberspace and websites have benefits such as increased awareness without having to leave home in the prevalence of coronavirus. Other benefits include easy information access, increasing attention and usefulness of information, high speed of information dissemination, access to information at low cost. Therefore, it is suggested that the website of medical universities should be introduced as a reliable and appropriate source to the general public.

According to the results of the evaluation of websites, it is suggested:

- To maintain a permanent page for health education on infectious diseases such as the coronavirus that shall be available from the main website of medical universities.
- Web-based databases should be created for information about academic and research resources related to coronavirus available for a wide public.

## Data Availability

The datasets used and analysed during the current study are available from the corresponding author upon reasonable request.

## Abbreviations

COVID-19: Coronavirus disease 2019
SPSS: Statistical package for social science
